# Explore the influencing factors and construct random forest models of post-stroke depression at 3 months in males and females

**DOI:** 10.1186/s12888-022-04467-0

**Published:** 2022-12-20

**Authors:** Xiuli Qiu, He Wang, Yan Lan, Jinfeng Miao, Chensheng Pan, Wenzhe Sun, Guo Li, Yanyan Wang, Xin Zhao, Zhou Zhu, Suiqiang Zhu

**Affiliations:** 1 Department of Neurology, Tongji Hospital, Tongji Medical College, Huazhong University of Science and Technology, 1095 Jiefang Avenue, Wuhan, 430030 Hubei China; 2 Department of Medical Affair, Tongji Medical College, Tongji Hospital, Huazhong University of Science and Technology, 1095 Jiefang Avenue, Wuhan, 430030 Hubei China

**Keywords:** Post-stroke depression, Random forest model, Sex difference

## Abstract

**Background:**

Post-stroke depression (PSD) is one of the most common neuropsychiatric complications after stroke. The occurrence, development and prognosis of PSD have long been different between males and females. The main purpose of this study was to explore the influencing factors of PSD at 3 months in males and females, and construct random forest (RF) models to rank the influencing factors.

**Methods:**

This is a prospective multicenter cohort study (Registration number: ChiCTR-ROC-17013993). Stroke patients hospitalized in the department of Neurology of three hospitals in Wuhan were enrolled from May 2018 to August 2019. Scale assessments were performed 24 hours after admission and 3 months after stroke onset. Binary logistic regression analysis was used for univariate and multivariate (stepwise backward method) analysis, when p was less than 0.05, the difference between groups was considered statistically significant. Lastly, the RF models were constructed according to the results of multivariate regression analysis.

**Results:**

This study found that several baseline variables were associated with PSD at 3 months in males and females. RF model ranked them as stroke severity (OR [odds ratio] =1.17, *p* < 0.001, 95%CI [confidence interval]:1.11–1.24), neuroticism dimension (OR = 1.06, *p* = 0.002, 95%CI:1.02–1.10), physical exercise (OR = 0.62, *p* = 0.007, 95%CI:0.44–0.88), sleeping time < 5 h (OR = 1.91, *p* = 0.006, 95% CI:1.20–3.04) and atrial fibrillation (OR = 4.18, *p* = 0.012, 95%CI:1.38–12.68) in males. In females, RF model ranked them as psychological resilience (OR = 0.98, *p* = 0.015, 95%CI:0.96–1.00), ability of daily living (OR = 0.98, *p* = 0.001, 95%CI:0.97–0.99), neuroticism dimension (OR = 1.11, *p* = 0.002, 95%CI:1.04–1.18) and subjective support (OR = 1.11, *p* < 0.001, 95%CI:1.05–1.78).

**Conclusion:**

The study found influencing factors of PSD at 3 months were different in males and females, and construct RF models to rank them according to their importance. This suggests that clinicians should focus their interventions on sex-specific influencing factors in order to improve the prognosis of PSD patients.

**Trial registration:**

ChiCTR-ROC-17013993.

**Supplementary Information:**

The online version contains supplementary material available at 10.1186/s12888-022-04467-0.

## Introduction

Post-stroke depression (PSD) is one of the most common neuropsychiatric complications after stroke [1]. According to a review, the incidence of PSD within 5 years after stroke was as low as 29% and as high as 52% [2]. The 10-year mortality rate of patients with PSD was 3.4 times higher than that of non-PSD [3]. Patients with PSD were more prone to social isolation and social defeat due to physical and psychological disorders [4]. One year after stroke, 15% of patients with PSD reported suicidal ideation [5].

The occurrence, development and prognosis of PSD have long been different between males and females [6–8]. Females need to focus on family life and social work, they may bear more stress. Many studies have found that the incidence of PSD in females is higher than that in males [9, 10], and some studies reported that the incidence of PSD in females is similar to or significantly lower than that in males [11–13]. Perhaps because of inconsistencies in sample size, race, or time of evaluation, there is no consistent conclusion about the sex difference in the incidence of PSD, this study explores this and hopes to provide reference for future research.

Previous studies have found that some associated sociodemographic factors differ in male and female PSD patients, such as age, cognitive impairment and physical exercise habits [14, 15]. In addition, previous literatures have reported that some hormones were associated with PSD, such as free T3, free T4, thyroid stimulating hormone (TSH) [16, 17], homocysteine [18], cortisol [19], brain derived neurotrophic factor (BDNF) [20], cortisol [21] and ACTH [22]. Only one study reported sex difference of blood biomarkers in PSD [15]. Herein, we plan to investigate the influence of sociodemographic and biochemical factors in PSD based on sex differences.

Machine learning algorithms have been widely used in the field of medicine and health, and random forest (RF) is one of the most commonly used machine learning algorithms. RF is often used to explore tumor markers [23], predict tumor prognosis [24], predict cardiovascular disease and postpartum depression risk [25, 26], etc. Therefore, the main purpose of this study was to explore the influencing factors of PSD at 3 months in males and females, and construct random forest (RF) models to rank the influencing factors.

## Methods

### Study population and design

This is a prospective multicenter cohort study (Registration number: ChiCTR-ROC-17013993). This protocol was approved by the Ethics Committee of Tongji Medical College, Huazhong University of Science and Technology (Approved No. of ethic committee: TJ-IRB20171108). A total of 891 stroke patients hospitalized in the department of Neurology of Tongji Hospital, Wuhan First Hospital and Wuhan Central Hospital in Wuhan, Hubei Province, China were enrolled from May 2018 to August 2019. In accordance with the Declaration of Helsinki, all subjects gave written informed consent [27].

The inclusion criteria for this study were as follows: (1) age ≥ 18 years; (2) hospitalized within 7 days after stroke onset (including hemorrhagic and ischemic stroke); (3) stroke was confirmed by computed tomography (CT) or magnetic resonance imaging (MRI) scan; (4) blood samples were collected within 24 hours after admission; (5) informed consent signed by patients or family members. Exclusion criteria were: (1) brain dysfunction caused by non-vascular diseases such as brain trauma, brain tumor and metastatic brain tumor; (2) have a history of anxiety, depression or other mental diseases or take related drugs; (3) aphasia (Boston Diagnostic Aphasia Examination grade less than or equal to 3), blindness, deafness and cognitive dysfunction (Mini-Mental State Examination score < 17 points); (4) subarachnoid hemorrhage; (5) unable to complete follow up. The inclusion and exclusion criteria were similar to our previous studies [27–29].

The study looked for factors associated with PSD at 3 months in males and females, with the severity of depressive symptoms assessed by the Hamilton Depression Scale-17 items (HAMD-17). PSD was diagnosed by a psychiatrist at 3 months after stroke onset. With the diagnostic criteria for PSD in the Diagnostic and Statistical Manual of Mental Disorders, 5th edition (DSM-V) (depression due to other medical conditions) being met and HAMD-17 score greater than 7 was used as the primary endpoint [30–33].

### Data collection

A standardized questionnaire was used to collect demographic and medical history information on patients within 24 hours of admission, including age, stroke type, education level, smoking history, drinking history, sleeping time < 5 h (Three or more days per week with less than 5 hours of sleep for more than a month), diabetes mellitus, hypertension, hyperlipidemia, atrial fibrillation, stroke history, physical exercise (WHO recommends that adults ages 18 to 64 complete at least 150 min of moderate-intensity aerobic physical activity a week, lasting at least 10 min each time, and muscle strength training two times a week). Venous blood samples were collected in the early morning of the second day (within 24 hours of admission) and sent to the laboratory for testing. Laboratory test indexes included free T3, free T4, TSH, homocysteine, cortisol, BDNF and adrenocorticotropic hormone (ACTH).

Eysenck Personality Questionnaire (EPQ), Connor—Davidson resilience scale (CD-RISC), National Institutes of Health Stroke Scale (NIHSS), Social Support Rating Scale (SSRS), Barthel index (BI) were assessed at admission and HAMD-17 was assessed all by two qualified and formally trained doctors (C.P. and W.S) at 3 months after stroke onset by clinic or WeChat. EPQ include four dimensions. Introversion-extroversion (E), scores range from 0 to 21 points, a high score indicates more extraversion. Neuroticism (N), scores range from 0 to 23 points, a high score indicates more anxiety and worried. Psychoticism (P), scores range from 0 to 24 points, a high score indicates more loneliness and apathy. Lie (L), scores range from 0 to 20 points, a high score indicates more masked affection [34]. CD-RISC include toughness, power and optimism, the total score of the scale is 100 points, higher scores indicate greater resilience to stress [35]. NIHSS is a relatively common scale in the world to evaluate the degree of neurological impairment in stroke patients. The total score is 42 points, and the higher the score indicates more serious neurological impairment [36]. SSRS include objective support, subjective support and use of support, the total score of the scale is 40 points, higher scores indicate more social support [37]. BI is the most widely used assessment method of daily living ability, with a total score of 100 points. The higher the score, the better the self-care ability [38].

### Statistical analysis

The Statistical Program for Social Sciences (SPSS) statistical software (version 25, Chicago, IL, USA) was used for data analysis. Categorical variables were represented by the number of cases and percentage. Continuous variables were represented by median and inter-quartile range (IQR) or mean ± standard deviation. The outcome variable was PSD at 3 months after stroke, coded as “Non-PSD” =0 or “PSD” =1. The total sample was divided into male group and female group for statistical analysis and model construction. Binary logistic regression analysis was used for univariate and multivariate (stepwise backward method) analysis, when p was less than 0.05, the difference between groups was considered statistically significant. Independent variables were checked for≥10 observations per outcome category. Besides, the independent variables included in the multivariate binary logistic regression analysis were diagnosed as collinearity. When the tolerance was greater than 0.2, the independent variables were considered to have no collinearity. Consistency between observers for HAMD-17 assessment was determined using intraclass correlation coefficient (ICC).

Multivariate binary logistic regression analysis models can only select statistically significant variables, but cannot distinguish the importance of variables. RF is robustness against overfitting, user-friendliness and the easy interpretation of the model. By constructing RF models, it is possible to rank the importance of variables so that the most important variables can be focused. The two RF models were developed using “rfPermute” package of R software (v4.0.0; http://www.r-project.org/). RF is a common machine learning model that constructs many decision trees and outputs the classes of a single tree (in the case of classification). The prediction result of the input instance will be determined by majority vote. Out-of-bag (OOB) refers to a dataset obtained by repeated sampling for training the decision tree every time a decision tree is established, and the remaining data is used to evaluate the performance of the decision tree and calculate the prediction error rate of the model, which is called OBB error. OOB errors were used to measure the performance of the model on the training set [23]. Males and females’ data were divided into training set (80%) and test set (20%), respectively. The training set was used to train the model with 100 trees, and the test set was used to validate the model.

The receiver operating characteristics (ROC) curve was plotted and the area under the curve (AUC) was calculated using the “pRoc” package of R software. AUC is used to evaluate the predictive performance of the model. It is generally believed that the prediction effect of the model is poor if the AUC value is less than 0.7; if the AUC value is greater than 0.7 but less than 0.9, the model has good prediction effect; when the AUC value is greater than 0.9, the prediction effect of the model is best but prone to overfitting.

## Results

**Fig. 1:**
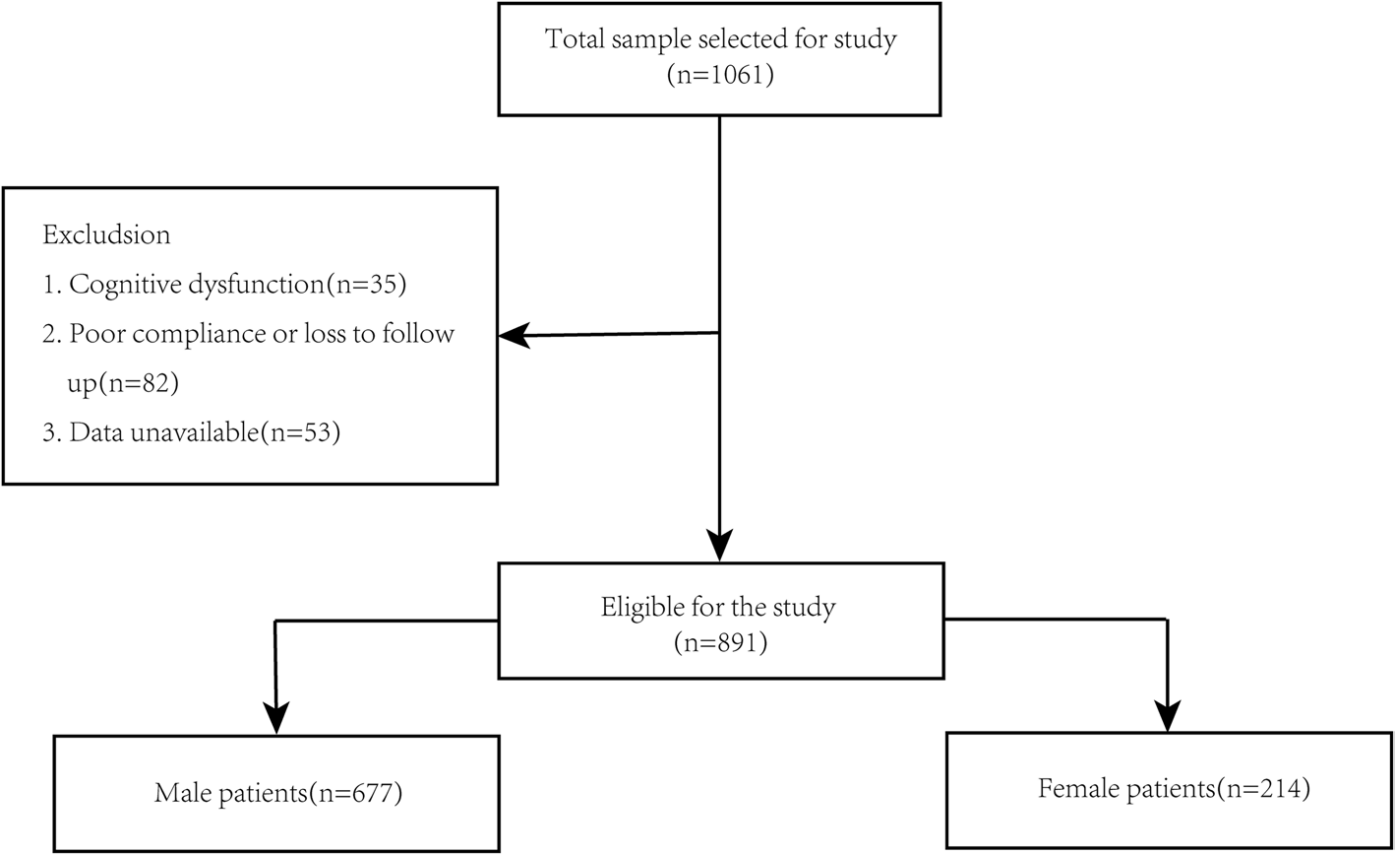
Flow chart

This study enrolled 891(male:677; female:214) stroke patients (Fig. 1. Flow chart). The mean age was 58.46 ± 10.12 of total patients. The proportion of cerebral infarction and hemorrhage was 88.0 and 12.0%, respectively. The mean age of males was 58.24 ± 10.91, and the proportion of cerebral infarction and hemorrhage was 90 and 10%, respectively. The mean age of females was 59.35 ± 11.26, and the proportion of cerebral infarction and hemorrhage was 83 and 17%, respectively. The measurements of HAMD-17 score (ICC = 0.92, 95%CI:0.79–0.97) had high interobserver consistency. The number of observations between the categories of independent and dependent variables was only atrial fibrillation < 10 patients. The incidence of PSD in males and females in this study was 37.7 and 47.2% (Chi^2^ = 6.16, *p* = 0.013), respectively.

Univariate binary logistic regression analysis found that the baseline variables associated with PSD in males at 3 months included education level (*p* = 0.025), sleeping time < 5 h (*p* = 0.011), atrial fibrillation (*p* = 0.002), physical exercise (*p* = 0.002), stroke severity (NIHSS score) (*p* < 0.001), ability of daily living (BI score) (*p* < 0.001), E dimension (*p* = 0.014), N dimension (*p* < 0.001), P dimension (*p* = 0.015), psychological resilience (CD-RISC score) (*p* = 0.005), free T3 (*p* = 0.003), cortisol (*p* = 0.029) and ACTH (*p* = 0.021) (Table 1). Baseline education level (*p* = 0.035), atrial fibrillation (*p* = 0.014), stroke severity (*p* = 0.001), BI score (*p* < 0.001), E dimension (*p* = 0.029), N dimension (*p* < 0.001), L dimension (*p* = 0.022), subjective support (*p* < 0.001), psychological resilience (*p* < 0.001), free T3 (*p* < 0.001), BDNF (*p* = 0.001) and ACTH (*p* = 0.029) were associated with PSD in females at 3 months (Table 2).

**Table 1:** Univariate binary logistic regression analysis for baseline demographic and biochemical variables of males

Variable	PSD (*N* = 255)	Non-PSD (*N* = 422)	βcoefficient	*p* value
Age, mean ± SD	58.8 ± 10.2	57.9 ± 11.3	0.01	0.295
Stroke type			0.25	0.344
Infarction, n(%)	227 (89.0)	385 (91.2)		
Hemorrhage, n(%)	28 (11.0)	37 (8.8)		
Education level				
Low, n(%)	63 (24.7)	81 (19.2)	Ref	
Medium, n(%)	157 (61.6)	251 (59.5)	−0.22	0.804
High, n(%)	35 (13.7)	90 (21.3)	−0.69	0.500
Smoking history, n(%)	183 (71.8)	316 (74.9)	−0.16	0.372
Drinking history, n(%)	89 (34.9)	136 (32.2)	0.12	0.474
**Sleeping time < 5 h, n(%)**	46 (18.0)	47 (11.1)	0.56	**0.011**
Diabetes Mellitus, n(%)	69 (27.1)	116 (27.5)	−0.02	0.903
Hypertension, n(%)	149 (58.4)	252 (59.7)	−0.05	0.742
Hyperlipidemia, n(%)	59 (23.1)	95 (22.5)	0.04	0.851
**Atrial fibrillation, n(%)**	13 (5.1)	5 (1.2)	1.50	**0.002**
Stroke history, n(%)	46 (18.0)	96 (22.7)	−0.29	0.145
**Physical exercise, n(%)**	79 (31.0)	182 (43.1)	−0.52	**0.002**
**NIHSS score, median (IQR)**	4.0 (2.0–7.0)	2.0 (1.0–4.0)	0.17	**< 0.001**
**BI score, median (IQR)**	80.0 (40.0–100.0)	95.0 (65.0–100.0)	− 0.02	**< 0.001**
EPQ				
**E dimension, median (IQR)**	11.0 (7.0–14.0)	12.0 (9.0–16.0)	−0.04	**0.014**
**N dimension, median (IQR)**	8.0 (6.0–13.0)	7.0 (4.0–11.0)	0.07	**< 0.001**
**P dimension, median (IQR)**	5.0 (4.0–7.0)	5.0 (3.0–7.0)	0.06	**0.015**
L dimension, median (IQR)	13.0 (11.0–15.0)	14.0 (12.0–15.0)	− 0.04	0.053
SSRS				
Objective support, median (IQR)	9.0 (7.0–11.0)	9.0 (7.0–11.0)	− 0.02	0.725
Subjective support, median (IQR)	23.0 (18.0–27.0)	22.0 (16.8–26.3)	0.03	0.081
Use of support, median (IQR)	7.0 (6.0–8.0)	7.0 (6.0–8.0)	−0.01	0.908
CD-RISC score, median (IQR)	64.0 (53.0–72.0)	65.0 (57.0–77.0)	− 0.02	0.005
**Free T3, median (IQR)**	2.5 (2.3–2.9)	2.7 (2.4–3.1)	−0.20	**0.003**
Free T4, median (IQR)	1.0 (0.9–1.1)	1.0 (0.9–1.1)	0.01	0.095
TSH, median (IQR)	1.6 (1.0–2.4)	1.7 (1.0–2.6)	−0.08	0.442
Homocysteine, median (IQR)	14.9 (12.4–18.0)	15.0 (12.1–19.4)	− 0.01	0.360
BDNF, median (IQR)	3.6 (2.1–6.9)	3.7 (2.1–7.4)	−0.02	0.962
**Cortisol, median (IQR)**	13.6 (11.0–16.9)	13.0 (10.4–15.9)	0.04	**0.029**
**ACTH, median (IQR)**	33.5 (20.6–48.0)	29.6 (15.4–46.9)	0.01	**0.021**

*NIHSS* National Institutes of Health Stroke Scale, *BI* Barthel index, *EPQ* Eysenck Personality Questionnaire, *E* Introversion-extroversion, *N* Neuroticism, *P* Psychoticism, *L* Lie, *SSRS* Social Support Rating Scale, *CD-RISC* Connor—Davidson resilience scale, *TSH* Thyroid Stimulating Hormone, *BDNF* Brain-Derived Neurotrophic Factor, *ACTH* Adrenocorticotropic Hormone

**Table 2:** Univariate binary logistic regression analysis for baseline demographic and biochemical variables of females

Variable	PSD (*n* = 101)	Non-PSD (*n* = 113)	βcoefficient	*p* value
Age, mean ± SD	58.8 ± 10.9	60.0 ± 11.6	− 0.01	0.277
Stroke type			0.07	0.846
Infarction, n(%)	83 (82.2)	94 (83.2)		
Hemorrhage, n(%)	18 (17.8)	19 (16.8)		
Education level				
Low, n(%)	56 (55.4)	55 (48.7)	Ref	
Medium, n(%)	40 (39.6)	40 (35.4)	−0.02	0.951
**High, n(%)**	5 (5.0)	18 (15.9)	−1.30	**0.016**
Smoking history, n(%)	7 (6.9)	11 (9.7)	−0.37	0.461
Drinking history, n(%)	8 (7.9)	7 (6.2)	0.26	0.621
Sleeping time < 5 h, n(%)	18 (17.8)	20 (17.7)	0.01	0.981
Diabetes Mellitus, n(%)	18 (17.8)	24 (21.2)	−0.22	0.530
Hypertension, n(%)	66 (65.3)	69 (61.1)	0.18	0.517
Hyperlipidemia, n(%)	21 (20.8)	26 (23.0)	−0.13	0.696
**Atrial fibrillation, n(%)**	7 (6.9)	0 (0.0)	21.39	**0.014**
Stroke history, n(%)	15 (14.9)	19 (16.8)	−0.15	0.695
Physical exercise, n(%)	34 (33.7)	45 (39.8)	−0.27	0.351
**NIHSS score, median (IQR)**	4.0 (2.0–8.0)	3.0 (1.0–5.0)	0.15	**0.001**
**BI score, median (IQR)**	60.0 (35.0–95.0)	95.0 (65.0–100.0)	−0.02	**< 0.001**
EPQ				
**E dimension, median (IQR)**	11.0 (7.0–14.0)	12.0 (9.0–15.0)	−0.07	**0.029**
**N dimension, median (IQR)**	11.0 (8.5–16.0)	7.0 (4.0–11.0)	0.14	**< 0.001**
P dimension, median (IQR)	4.0 (3.0–6.0)	4.0 (3.0–6.0)	0.01	0.912
**L dimension, median (IQR)**	14.0 (12.0–16.0)	15.0 (12.0–17.0)	−0.10	**0.022**
SSRS				
Objective support, median (IQR)	10.0 (8.0–11.0)	9.0 (8.0–11.0)	−0.02	0.940
**Subjective support, median (IQR)**	25.0 (20.0–28.0)	22.0 (16.0–25.0)	0.09	**< 0.001**
Use of support, median (IQR)	7.0 (5.0–8.0)	7.0 (6.0–8.5)	−0.10	0.109
**CD-RISC score, median (IQR)**	55.0 (43.0–64.5)	62.0 (52.0–75.5)	−0.03	**0.001**
**Free T3, median (IQR)**	2.3 (2.1–2.6)	2.6 (2.3–3.1)	− 0.15	**< 0.001**
Free T4, median (IQR)	1.0 (1.0–1.2)	1.1 (0.9–10.1)	−0.16	0.246
TSH, median (IQR)	2.1 (1.1–2.9)	2.1 (1.2–3.4)	−0.10	0.495
Homocysteine, median (IQR)	10.9 (9.2–13.6)	12.0 (10.4–13.9)	−0.04	0.102
**BDNF, median (IQR)**	2.9 (1.7–4.5)	5.0 (2.4–10.7)	−0.01	**0.001**
Cortisol, median (IQR)	13.9 (10.5–17.6)	13.1 (10.5–15.6)	0.04	0.197
**ACTH, median (IQR)**	24.8 (17.7–41.2)	22.8 (9.6–36.8)	0.01	**0.029**

*NIHSS* National Institutes of Health Stroke Scale, *BI* Barthel index, *EPQ* Eysenck Personality Questionnaire, *E* Introversion-extroversion, *N* Neuroticism, *P* Psychoticism, *L* Lie, *SSRS* Social Support Rating Scale, *CD-RISC* Connor—Davidson resilience scale, *TSH* Thyroid Stimulating Hormone, *BDNF* Brain-Derived Neurotrophic Factor, *ACTH* Adrenocorticotropic Hormone

Collinearity diagnosis showed that there was no collinearity between the independent variables (Table S3 and Table S4). Multivariate binary logistic regression analysis found that the baseline variables associated with PSD in males at 3 months included sleeping time < 5 h (OR = 1.91, *p* = 0.006, 95%CI:1.20–3.04), atrial fibrillation (OR = 4.18, *p* = 0.012, 95%CI:1.38–12.68), physical exercise (OR = 0.62, *p* = 0.007, 95%CI:0.44–0.88), stroke severity (OR = 1.17, *p* < 0.001, 95%CI:1.11–1.24) and N dimension (OR = 1.06, *p* = 0.002, 95%CI:1.02–1.10) (Table 2). Baseline ability of daily living (OR = 0.98, *p* = 0.001, 95%CI:0.97–0.99), N dimension (OR = 1.11, *p* = 0.002, 95%CI:1.04–1.18), subjective support (OR = 1.11, p < 0.001, 95%CI:1.05–1.78) and psychological resilience (OR = 0.98, *p* = 0.015, 95%CI:0.96–1.00) were associated with PSD in females at 3 months (Table 3).

**Table 3:** Multivariate binary logistic regression analysis for baseline variables of males and females

Variables	βcoefficient	OR	95%CI	*p* value
Male				
Sleeping time < 5 h	0.67	1.96	1.23 ~ 3.12	0.005
Atrial fibrillation	1.47	4.35	1.44 ~ 13.13	0.009
Physical exercise	−0.44	0.64	0.45 ~ 0.92	0.015
NIHSS score	0.15	1.17	1.11 ~ 1.23	< 0.001
E dimension	−0.04	0.97	0.93 ~ 1.00	0.063
N dimension	0.06	1.06	1.02 ~ 1.10	0.002
Cortisol	0.03	1.03	1.00 ~ 1.07	0.078
Female				
Education level				
Low		Ref		
Medium	0.13	1.14	0.57 ~ 2.26	0.718
High	−1.17	0.31	0.09 ~ 1.04	0.058
Subjective support	0.10	1.11	1.05 ~ 1.17	0.001
BI score	−0.02	0.98	0.97~0.99	0.001
N dimension	0.11	1.12	1.05 ~ 1.20	0.001
CD-RISC score	−0.02	0.98	0.96 ~ 1.00	0.017

*NIHSS* National Institutes of Health Stroke Scale, *N* Neuroticism, *E* Introversion-extroversion, *BI* Barthel index, *CD-RISC* Connor—Davidson resilience scale; A negative beta coefficient represents a protective factor; A positive beta coefficient represents a risk factor

**Fig. 2:**
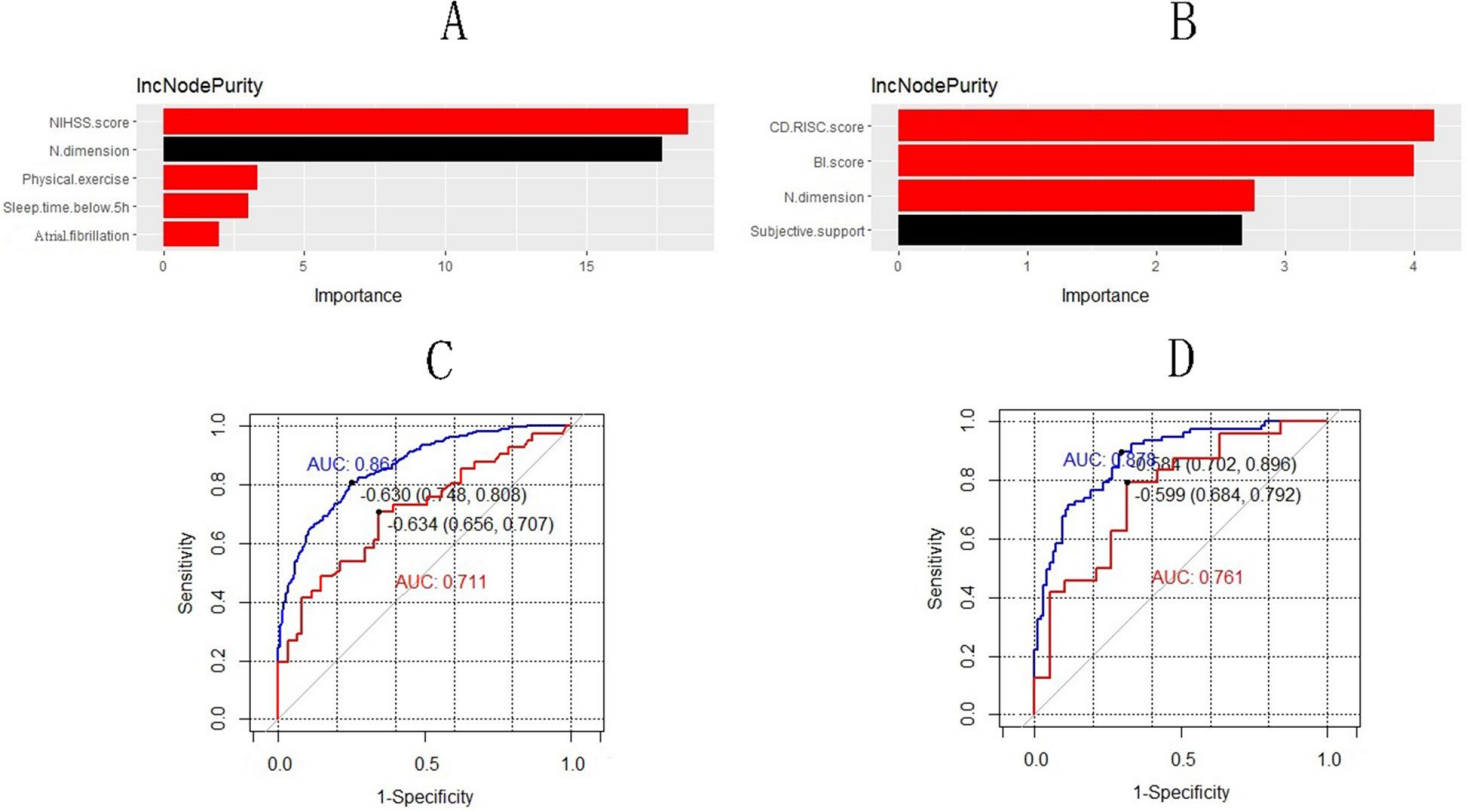
**A** The order of importance of baseline variables influencing PSD in males at 3 months; NIHSS: National Institutes of Health Stroke Scale; N: Neuroticism; The red column represented a statistically significant difference which was tested by permutation test. **B** The order of importance of baseline variables influencing PSD in females at 3 months; BI: Barthel index; N: Neuroticism; CD-RISC: Connor—Davidson resilience scale; The red column represented a statistically significant difference which was tested by permutation test. **C** Receiver operator characteristic (ROC) curves and area under the curve (AUC) values for the training group (blue line) and validation group (red line) of the males. **D** ROC curves and AUC values for the training group (blue line) and validation group (red line) of the females

RF models were constructed based on the results of multivariate binary logistic regression analysis. The order of importance of baseline variables influencing PSD in males at 3 months was stroke severity, N dimension, physical exercise, sleeping time < 5 h and atrial fibrillation. The order of importance of baseline variables influencing PSD in females at 3 months was psychological resilience, ability of daily living, N dimension and subjective support. For male patients, the AUC values of the training set and the test set were 0.86 (95%CI:0.83–0.89) and 0.71 (95%CI:0.61–0.82), respectively (Fig. 2; Table 3). For female patients, the AUC values of the training set and the test set were 0.88 (95%CI:0.83–0.93) and 0.76 (95%CI:0.61–0.91), respectively (Fig. 2; Table 4).

**Table 4:** Performance comparison of the males and females model in training and validation group

	Training group		Validation group	
	Male	Female	Male	Female
AUC	0.86	0.88	0.71	0.76
Sensitivity	0.75	0.90	0.66	0.79
Specificity	0.81	0.70	0.71	0.68
Accuracy	0.77	0.79	0.68	0.74
Cut-off value	−0.63	−0.58	−0.63	−0.60

*AUC* area under the curve

## Discussion

This is a prospective cohort study suggesting that the influencing factors of PSD at 3 months were different in males and females. The order of importance of baseline variables influencing PSD at 3 months was stroke severity, neuroticism dimension, physical exercise, sleeping time < 5 h and atrial fibrillation in males; psychological resilience, ability of daily living, neuroticism dimension and subjective support in females. The sex difference in risk and influencing factors should be focused on in the prevention and treatment of PSD.

Because previous studies have found and reported the relationship between sleeping time, atrial fibrillation, physical exercise, stroke severity, neuroticism dimension, subjective support, ability of daily living, psychological resilience and PSD [27, 39–41], this study focused on whether there were sex differences in these factors, and did not explain the factors one by one. As for the ability of daily living, it was not significant in multivariate binary logistic regression analysis in males, we did a stratified analysis and found that the ability of daily living was associated with PSD only in the moderate-severe stroke group (Table S5).

In this study, the incidence of PSD in the total sample, males, females were 40.0, 37.7, 47.2%, respectively. They were lower than those reported in a foreign study (54.8, 48.0, 65.0%) [42], possibly because the foreign study reported self-reported depression and classified sometimes depressed patients as PSD. However, they were higher than other domestic studies (28.2, 27.9, 43.5%) [43, 44], possibly because these studies excluded patients with renal insufficiency and the proportion of loss to follow-up was more than ours. Still, the results are similar to those of other study [45].

The RF model was used to rank the baseline variables influencing PSD in males at 3 months as stroke severity, neuroticism dimension, physical exercise, sleeping time < 5 h and atrial fibrillation. It suggests that clinicians should pay attention to the importance of influencing factors in the prevention and treatment of PSD in males. Firstly, actively promote recovery of limb function, as better recovery of limb function can alleviate depressive symptoms [40, 41]. Secondly, conduct personality test screening and paying more attention to the patients with higher neuroticism dimension scores who are more likely to have depressive symptoms. Thirdly, patients are encouraged to do proper physical exercise and keep adequate sleep. Lastly, actively treat atrial fibrillation and other cardiovascular diseases.

The RF model was used to rank the baseline variables influencing PSD in females at 3 months as psychological resilience, ability of daily living, neuroticism dimension and subjective support. For the treatment and prevention of PSD in females, clinicians should pay attention to the influencing factors according to their importance. Clinicians should focus first on psychological resilience, then on ability of daily living, then on personality, and finally on subjective support. In addition to the routine application of antidepressants and promotion of limb function recovery, clinicians could integrate psychological intervention therapy to help patients establish the right mindset. For example, personality, social support and psychological resilience should be evaluated for stroke patients, and cognitive behavioral therapy or family therapy should be conducted for high-risk patients.

This study has several advantages. First, it is a prospective multicenter cohort study with high reliability. Second, because males and females are so different, most previous studies treated them as a whole and may have overlooked some individual factors. In this study, males and females were separately analyzed to help find the influencing factors of PSD based on sex. Lastly, few Chinese studies have focused specifically on the risk of PSD in females. This study conducted a separate analysis on females and found the influencing factors of PSD in females.

Some limitations to our study must also be noted. First, because only internal validation was performed, and the sample size of females is smaller than that of males, resulting in lower precise of female RF model. Second, the follow-up period was short, only 3 months, and a longer follow-up period should be conducted to better observe the incidence of depressive symptoms. Third, patients with aphasia, history of depression, blindness, deafness, and cognitive impairment were excluded from the study, so the proportion of mood disorders may be underestimated. Finally, the number of observations of atrial fibrillation was < 10 patients, further sample size expansion may be necessary to increase reliability.

## Conclusion

The study found influencing factors of PSD at 3 months were different in males and females, and construct RF models to rank them according to their importance. This suggests that clinicians should focus their interventions on sex-specific influencing factors in order to improve the prognosis of PSD patients.

## Supplementary Information

**Additional file 1: Table S1.** The comparison of demographic and clinical variables between training group and validation group of male and female patients. **Table S2.** The comparison of demographic variables in PSD with and without antidepressant use of male and female patients. **Table S3.** Tolerance of collinearity diagnosis between independent variables entered into binary logistic regression analyses for males. **Table S4.** Tolerance of collinearity diagnosis between independent variables entered into binary logistic regression analyses for female. **Table S5.** The association between BI score and PSD at 3 months in males according to stroke severity. **Figure S1.** A: The number of male random forest trees; B: The number of female random forest trees.

## Data Availability

The datasets presented in this article are not readily available because further data mining is ongoing. Requests to access the datasets should be directed to 15738862357@163.com. The R codes of RF model are available in the supplementary material.
