# Supplementary material for: Explore the influencing factors and construct random forest models of post-stroke depression at 3 months in males and females
Source: BMC Psychiatry. 2022 Dec 20;22:811. doi: 10.1186/s12888-022-04467-0 (PMC9764471; doi:10.1186/s12888-022-04467-0)
Supplement: Supplementary file 1 — Additional file 1: Table S1. The comparison of demographic and clinical variables between training group and validation group of male and female patients. Table S2. The comparison of demographic variables in PSD with and without antidepressant use of male and female patients. Table S3. Tolerance of collinearity diagnosis between independent variables entered into binary logistic regression analyses for males. Table S4. Tolerance of collinearity diagnosis between independent variables entered into binary logistic regression analyses for female. Table S5. The association between BI score and PSD at 3 months in males according to stroke severity. Figure S1. A: The number of male random forest trees; B: The number of female random forest trees. [file 12888_2022_4467_MOESM1_ESM.docx]

Table S1. The comparison of demographic and clinical variables between training group and validation group of male and female patients.

| Variable | Male | | | Female | | |
| --- | --- | --- | --- | --- | --- | --- |
|  | Training group(n=542) | Validation group(n=135) | p value | Training group(n=171) | Validation group(n=43) | p value |
| Age, mean±SD | 58.3±11.0 | 58.0±10.7 | 0.768 | 59.6±11.0 | 58.4±12.2 | 0.515 |
| Stroke type  Infarction, n(%)  Hemorrhage, n(%) | 491(90.6)  51(9.4) | 121(89.6)  14(10.4) | 0.735 | 137(80.1)  34(19.9) | 40(90.3)  3(7.0) | 0.045 |
| Education level  Low, n(%)  Medium, n(%)  High, n(%) | 115(21.2)  327(60.3)  100(18.5) | 29(21.5)  81(60.0)  25(18.5) | 0.997 | 88(51.5)  61(35.7)  22(12.9) | 23(53.5)  19(44.2)  1(2.3) | 0.063 |
| Smoking history, n(%) | 408(75.3) | 91(67.4) | 0.063 | 14(8.2) | 4(9.3) | 1.000 |
| Drinking history, n(%) | 183(33.8) | 42(31.1) | 0.558 | 14(8.2) | 1(2.3) | 0.312 |
| Sleeping time＜5h, n(%) | 78(14.4) | 15(11.1) | 0.322 | 33(19.3) | 5(11.6) | 0.239 |
| Diabetes Mellitus, n(%) | 146(26.9) | 39(28.9) | 0.649 | 36(21.1) | 6(14.0) | 0.295 |
| Hypertension, n(%) | 321(59.2) | 80(59.3) | 0.994 | 109(63.7) | 26(60.5) | 0.691 |
| Hyperlipidemia, n(%) | 123(22.7) | 31(23.0) | 0.947 | 40(23.4) | 7(16.3) | 0.314 |
| Atrial fibrillation, n(%) | 16(3.0) | 2(1.5) | 0.515 | 6(3.5) | 1(2.3) | 0.679 |
| Stroke history, n(%) | 110(20.3) | 32(23.7) | 0.384 | 31(18.1) | 3(7.0) | 0.074 |
| Physical exercise, n(%) | 209(38.6) | 52(38.5) | 0.993 | 69(40.4) | 10(23.3) | 0.038 |
| NIHSS score, median(IQR) | 3(1-5) | 2(1-5) | 0.133 | 3(1-6) | 3(2-6) | 0.662 |
| BI score, median(IQR) | 90(58.75-100) | 95(60-100) | 0.320 | 80(40-100) | 90(45-100) | 0.298 |
| EPQ  E dimension, median(IQR)  N dimension, median(IQR)  P dimension, median(IQR)  L dimension, median(IQR) | 11(8-15)  8(5-11)  5(3-7)  13(11-15) | 12(8-15)  8(4-12)  5(3-7)  13(11-16) | 0.924  0.829  0.802  0.925 | 11(8-14)  10(6-15)  4(3-6)  14(12-16) | 10(7-14)  9(5-12)  4(3-5)  14(13-16) | 0.219  0.107  0.977  0.222 |
| SSRS  Objective support, median(IQR)  Subjective support, median(IQR)  Use of support, median(IQR) | 9(7-11)  22(17-27)  7(6-8) | 9(8-11)  23(18-27)  7(6-8) | 0.864  0.120  0.432 | 10(8-11)  23(18-27)  7(6-8) | 10(8-11)  23(16-28)  7(5-8) | 0.936  0.504  0.201 |
| CD-RISC score, median(IQR) | 65(55-76) | 65(55-76) | 0.372 | 59(47-71) | 57(49-71) | 0.555 |
| Free T3, median(IQR) | 2.6(2.3-3.0) | 2.7(2.4-3.0) | 0.201 | 2.5(2.1-2.7) | 2.5(2.2-3.0) | 0.231 |
| Free T4, median(IQR) | 1.0(0.9-1.1) | 1.0(0.9-1.1) | 0.963 | 1.0(0.9-1.3) | 1.1(10.-1.3) | 0.143 |
| TSH, median(IQR) | 1.6(1.0-2.4) | 1.8(1.2-2.7) | 0.038 | 2.1(1.1-3.2) | 1.9(1.0-3.4) | 0.741 |
| Homocysteine, median(IQR) | 15.0(12.4-18.9) | 14.5(12.3-19.0) | 0.929 | 11.6(9.5-13.7) | 12.0(9.9-13.5) | 0.481 |
| BDNF, median(IQR) | 3.6(2.1-7.3) | 3.8(2.3-7.5) | 0.384 | 3.5(2.0-7.2) | 3.2(2.1-7.3) | 0.918 |
| Cortisol, median(IQR) | 13.2(10.4-16.2) | 13.0(11.1-16.8) | 0.554 | 13.7(10.5-16.2) | 13.2(10.7-15.3) | 0.376 |
| ACTH, median(IQR) | 29.9(17.7-47.2) | 36.1(19.8-48) | 0.101 | 23.7(14.3-39.8) | 20.2(11.3-35.6) | 0.283 |

NIHSS: National Institutes of Health Stroke Scale; BI: Barthel index; EPQ: Eysenck Personality Questionnaire; E: Introversion-extroversion; N: Neuroticism; P: Psychoticism; L: Lie; SSRS: Social Support Rating Scale;

CD-RISC: Connor—Davidson resilience scale; TSH: Thyroid Stimulating Hormone; BDNF: Brain-Derived Neurotrophic Factor; ACTH: Adrenocorticotrophic hormone

Table S2. The comparison of demographic variables in PSD with and without antidepressant use of male and female patients.

| Variable | Male | | | Female | | |
| --- | --- | --- | --- | --- | --- | --- |
|  | PSD with antidepressant (n=38) | PSD without antidepressant (n=280) | p value | PSD with antidepressant (n=21) | PSD without antidepressant (n=97) | p value |
| Age, mean±SD | 56.9±10.7 | 57.6±10.6 | 0.762 | 58.0±8.0 | 58.0±11.9 | 0.781 |
| Stroke type  Infarction, n(%)  Hemorrhage, n(%) | 34(89.5)  4(10.5) | 246(87.9)  34(12.1) | 0.983 | 17(81.0)  4(19.0) | 75(77.3)  22(22.7) | 0.941 |
| Education level  Low, n(%)  Medium, n(%)  High, n(%) | 10(26.3)  22(57.9)  6(15.8) | 70(25)  167(59.6)  43(15.4) | 0.978 | 13(61.9)  5(23.8)  3(14.3) | 52(53.6)  39(40.2)  6(6.2) | 0.229 |
| Smoking history, n(%) | 26(68.4) | 206(73.6) | 0.502 | 2(9.5) | 6(6.2) | 0.942 |
| Drinking history, n(%) | 14(36.8) | 95(33.9) | 0.723 | 5(23.8) | 6(6.2) | 0.035 |
| Sleeping time＜5h, n(%) | 4(10.5) | 42(15.0) | 0.462 | 4(19.0) | 20(20.6) | 0.871 |
| Diabetes Mellitus, n(%) | 12(31.6) | 70(25.0) | 0.384 | 2(9.5) | 15(15.5) | 0.719 |
| Hypertension, n(%) | 24(63.2) | 154(55.0) | 0.342 | 14(66.7) | 59(60.8) | 0.617 |
| Hyperlipidemia, n(%) | 13(34.2) | 56(20.0) | 0.046 | 7(33.3) | 18(18.6) | 0.227 |
| Atrial fibrillation, n(%) | 3(7.9) | 9(3.2) | 0.333 | 2(9.5) | 4(4.1) | 0.636 |
| Stroke history, n(%) | 7(18.4) | 65(23.2) | 0.508 | 5(23.8) | 12(12.4) | 0.312 |
| Physical exercise, n(%) | 12(31.6) | 88(31.4) | 0.985 | 5(23.8) | 34(35.1) | 0.321 |
| NIHSS score, median(IQR) | 5.5(3.0-8.25) | 4(2-7) | 0.047 | 5(3.5-9) | 4(2-8) | 0.168 |
| BI score, median(IQR) | 57.5(35-85) | 78(40-100) | 0.028 | 40(25-80) | 60(35-90) | 0.112 |
| EPQ  E dimension, median(IQR)  N dimension, median(IQR)  P dimension, median(IQR)  L dimension, median(IQR) | 12(8.75-15.25)  10(6.75-14)  5(2.75-7)  14(11-16) | 11(7-14)  8.5(6-12)  5(3-7)  13(11-15) | 0.329  0.065  0.874  0.580 | 13(7.5-15)  11(6-16)  4(3-5)  14(12-16) | 11(7-13)  11(9-16)  4(3-6)  14(12.5-15) | 0.184  0.546  0.757  0.861 |
| SSRS  Objective support, median(IQR)  Subjective support, median(IQR)  Use of support, median(IQR) | 9.5(6-12)  23(18-29)  6(5-7.25) | 9(8-11)  22(18.25-27)  7(6-8) | 0.623  0.286  0.294 | 9(7.5-10.5)  23(19-26)  7(6-8) | 9(8-11)  27(22-29)  7(6-8) | 0.983  0.010  0.983 |
| CD-RISC score, median(IQR) | 63(54.75-78) | 65(53.25-73) | 0.728 | 53(45.5-67) | 56(43.5-65.5) | 0.704 |
| Baseline HAMD-17 score, median(IQR) | 15.5(14-20) | 12(10-16) | ＜0.001 | 19(14-20.5) | 14(10.5-18) | 0.012 |
| 3 months HAMD-17 score, median(IQR) | 10.5(8-14) | 8(5-11) | 0.011 | 11(5-16.5) | 11(7-14.5) | 0.849 |
| HAMD-17 score decreased(from baseline to 3 months), n(%) | 33(86.8) | 233(83.2) | 0.570 | 17(81.0) | 76(78.4) | 0.791 |

NIHSS: National Institutes of Health Stroke Scale; BI: Barthel index; EPQ: Eysenck Personality Questionnaire; E: Introversion-extroversion; N: Neuroticism; P: Psychoticism; L: Lie; SSRS: Social Support Rating Scale; CD-RISC: Connor—Davidson resilience scale;

Table S3. Tolerance of collinearity diagnosis between independent variables entered into binary logistic regression analyses for males

| Variables | Atrial fibrillation | Physical exercise | NIHSS score | BI score | E dimension | N dimension | P dimension | Free T3 | Cortisol | ACTH |
| --- | --- | --- | --- | --- | --- | --- | --- | --- | --- | --- |
| Sleeping time<5h | 0.98 | 0.94 | 0.50 | 0.50 | 0.94 | 0.90 | 0.87 | 0.97 | 0.89 | 0.90 |
| Atrial fibrillation |  | 0.94 | 0.50* | 0.50 | 0.94 | 0.90* | 0.87 | 0.97 | 0.89 | 0.90 |
| Physical exercise |  |  | 0.51* | 0.50 | 0.96*** | 0.90 | 0.88** | 0.97 | 0.89 | 0.90 |
| NIHSS score |  |  |  | 0.95*** | 0.96 | 0.90 | 0.89 | 0.97 | 0.90 | 0.91 |
| BI score |  |  |  |  | 0.97 | 0.91** | 0.89* | 0.99*** | 0.91** | 0.91 |
| E dimension |  |  |  |  |  | 0.91 | 0.92*** | 0.99 | 0.91 | 0.91 |
| N dimension |  |  |  |  |  |  | 1.00*** | 0.99 | 0.91 | 0.91 |
| P dimension |  |  |  |  |  |  |  | 0.99 | 0.91 | 0.91 |
| Free T3 |  |  |  |  |  |  |  |  | 0.91 | 0.91 |
| Cortisol |  |  |  |  |  |  |  |  |  | 1.00*** |

*p<0.05; **p<0.01; ***p<0.001; When the tolerance was greater than 0.2, the independent variables were considered to have no collinearity.

Table S4. Tolerance of collinearity diagnosis between independent variables entered into binary logistic regression analyses for female

| Variables | Atrial fibrillation | NIHSS score | BI score | E dimension | N dimension | L dimension | Subjective support | CD-RISC score | Free T3 | BDNF | ACTH |
| --- | --- | --- | --- | --- | --- | --- | --- | --- | --- | --- | --- |
| Education level | 0.90 | 0.41 | 0.40* | 0.84* | 0.75 | 0.79 | 0.84 | 0.76 | 0.95 | 0.93 | 0.88 |
| Atrial fibrillation |  | 0.41 | 0.40 | 0.84 | 0.77* | 0.82* | 0.84 | 0.76 | 0.95 | 0.93 | 0.91** |
| NIHSS score |  |  | 0.92*** | 0.85 | 0.77 | 0.82 | 0.86* | 0.76 | 0.95 | 0.93 | 0.92 |
| BI score |  |  |  | 0.85 | 0.76 | 0.82 | 0.89* | 0.76 | 0.96 | 0.93 | 0.92 |
| E dimension |  |  |  |  | 0.78 | 0.82 | 0.91* | 0.82*** | 0.96 | 0.93 | 0.92 |
| N dimension |  |  |  |  |  | 0.90*** | 0.92 | 0.89*** | 0.99* | 0.94 | 0.92 |
| L dimension |  |  |  |  |  |  | 0.93 | 0.95*** | 0.99 | 0.97* | 0.94 |
| Subjective support |  |  |  |  |  |  |  | 0.97* | 0.99 | 0.97 | 0.99** |
| CD-RISC score |  |  |  |  |  |  |  |  | 0.99 | 0.99* | 0.99 |
| Free T3 |  |  |  |  |  |  |  |  |  | 0.99 | 0.99 |
| BDNF |  |  |  |  |  |  |  |  |  |  | 1.00 |

*p<0.05; **p<0.01; ***p<0.001; When the tolerance was greater than 0.2, the independent variables were considered to have no collinearity.

Stroke was classified into five categories according to the NIHSS score used for National Institute of Neurological Disorders and Stroke (NINDS). Normal or nearly normal stroke: 0-1 score; Mild stroke: 2-4 score; Moderate stroke: 5-15 score; Moderate-Severe stroke: 16-20 score; Severe stroke: 21-42 score. In the male sample, only three patients belonged to moderate-severe stroke, and no patient belonged to severe stroke.

Table S5. The association between BI score and PSD at 3 months in males according to stroke severity

|  |  | PSD(n=255) | Non-PSD(n=422) | p value |
| --- | --- | --- | --- | --- |
| Nearly normal stroke group(n=203) | BI score | 100.00(90.00-100.00) | 100.00(96.25-100.00) | 0.085 |
| Mild stroke group(n=263) | BI score | 95.00(75.00-100.00) | 97.50(75.00-100.00) | 0.371 |
| Moderate stroke group(n=208) | BI score | 40.00(30.00-65.00) | 55.00(35.00-79.50) | 0.013 |

| A  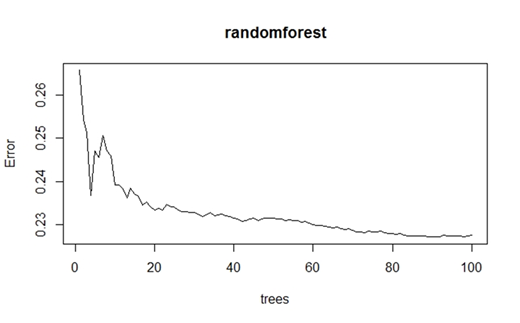 | B  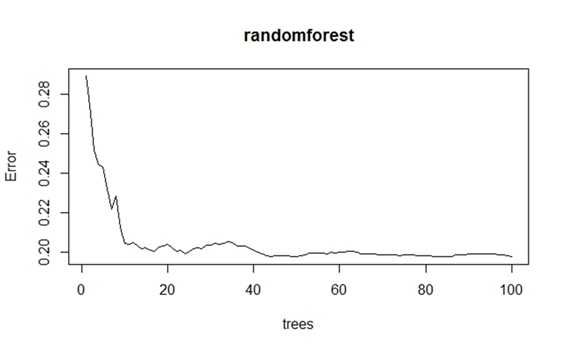 |
| --- | --- |

Figure S1. A: The number of male random forest trees; B: The number of female random forest trees.

As the number of trees increases, the stability of the model becomes better and better.

Random forest model R code for female, male R code is similar to female.

setwd("G:\\data")

mydata<-read.csv("data",header=T)

summary(mydata)

set.seed(1234)

prop_train <- 0.8

train <- sample(nrow(mydata), nrow(mydata)* prop_train)

trainset <- mydata[train, ]

testset <- mydata[-train, ]

dim(trainset)

dim(testset)

library(rfPermute)

set.seed(1234)

rf.train<-rfPermute(Depression~., data=trainset,ntree=100,mtry=2,proximity=TRUE,na.action=na.omit,importance=TRUE,nrep=100)

rf.train

print(rf.train)

plot(rf.train,main="randomforest")

importance_rf<-data.frame(importance(rf.train,scale=TRUE),check.names = FALSE)

importance_rf

summary(rf.train)

importance_rf.pval<-(rf.train$pval)[,,2]

importance_rf.pval

plot(rp.importance(rf.train,scale = TRUE))
